# 11039 Indiana CTSI High-School STEM Summer Research Program: Future opportunities from a 2020 virtual program

**DOI:** 10.1017/cts.2021.565

**Published:** 2021-03-30

**Authors:** Elmer Sanders, Leigh-Ann Cruz, Emily Speidell, Rose Schnabel, Adhitya Balaji, Elise Hogarth, Jade Miller, Sofia Vaides, Matthew R. Allen

**Affiliations:** 1 Indiana University School of Medicine; 2 Riverside High School; 3 Decatur Central High School; 4 Indiana University; 5 Purdue University; 6 University of Alabama; 7 Indiana University, Purdue University, of Indianapolis

## Abstract

ABSTRACT IMPACT: o The Indiana Clinical and Translational Sciences Institute K-12 STEM Outreach Program’s pivoted to a virtual program in summer 2020 which yielded novel approaches that could be retained in future years to extend the reach/impact of our pipeline program. OBJECTIVES/GOALS: o Provide students with a meaningful and safe research experience during the COVID Pandemic. o Develop new modules and approaches that could be delivered virtually. o Engage students from communities that were not possible in previous years when in person meetings were required. METHODS/STUDY POPULATION: o The program has historically supported over 100 high school students per year in a summer research internship for the last 5 years. Students are placed with academic research mentors in various Schools and Departments across the IUPUI campus, and also with industry laboratories. o COVID-related restrictions required development of 100% virtual program. Key aspects of the virtual program included: cohort-based research mentor assignments with 1-4 mentees matched per research mentor, research projects that could be conducted virtually, heavy engagement of high-school teachers to facilitate the research experience with cohorts of mentees, a more rigorous virtual seminar series that included new modules such as COVID-specific programming and thus enhancing public education about COVID. RESULTS/ANTICIPATED RESULTS: o The program served 130 students in summer 2020. o We were able to recruit new faculty and industry mentors involved in data science research. As a result, we have now increased our mentor pool to serve more students in the future. o Because student participation was virtual, we were able to accept students from further distances (up to 120 miles away) across the state. We were also able to accept local economically disadvantaged students that may have not been able to participate because of lack of reliable transportation. o A positive unanticipated outcome was that mentees relationships with the mentors was established virtually thus increasing the potential for students to remain engaged in their research. DISCUSSION/SIGNIFICANCE OF FINDINGS: o Adapting to a virtual platform provided research experience to high school students during a time when traditional approaches were not possible. Given some research experiences do not require in-person activities, this newly established model could be used moving forward to allow more statewide engagement in research experiences.

